# Interrelationship between Sleep and Exercise: A Systematic Review

**DOI:** 10.1155/2017/1364387

**Published:** 2017-03-26

**Authors:** Brett A. Dolezal, Eric V. Neufeld, David M. Boland, Jennifer L. Martin, Christopher B. Cooper

**Affiliations:** ^1^Exercise Physiology Research Laboratory, Departments of Medicine and Physiology, David Geffen School of Medicine at UCLA, Los Angeles, CA, USA; ^2^VA Greater Los Angeles Healthcare System, Geriatric Research, Education and Clinical Center, North Hills, CA, USA; ^3^Department of Medicine, David Geffen School of Medicine at UCLA, Los Angeles, CA, USA

## Abstract

Although a substantial body of literature has explored the relationship between sleep and exercise, comprehensive reviews and definitive conclusions about the impact of exercise interventions on sleep are lacking. Electronic databases were searched for articles published between January 2013 and March 2017. Studies were included if they possessed either objective or subjective measures of sleep and an exercise intervention that followed the guidelines recommended by the American College of Sports Medicine. Thirty-four studies met these inclusion criteria. Twenty-nine studies concluded that exercise improved sleep quality or duration; however, four found no difference and one reported a negative impact of exercise on sleep. Study results varied most significantly due to participants' age, health status, and the mode and intensity of exercise intervention. Mixed findings were reported for children, adolescents, and young adults. Interventions conducted with middle-aged and elderly adults reported more robust results. In these cases, exercise promoted increased sleep efficiency and duration regardless of the mode and intensity of activity, especially in populations suffering from disease. Our review suggests that sleep and exercise exert substantial positive effects on one another; however, to reach a true consensus, the mechanisms behind these observations must first be elucidated.

## 1. Introduction

Despite the overwhelming consensus that both sufficient sleep and adequate exercise are pivotal in maintaining health, these behaviors are often deprioritized within the typical American lifestyle. For example, the Centers for Disease Control and Prevention estimate that nearly one-third of adults sleep less than the recommended seven hours per night needed to maintain optimal health [1, 2]. An even larger sleep deficit is observed in teenagers: roughly two-thirds of high-school students, who are advised to sleep eight to ten hours, receive less than eight on school nights [3, 4]. Chronic sleep deprivation has been shown to increase the risk for a host of physical and mental illnesses as well as play a dominant role in motor vehicle accidents [5, 6]. Furthermore, poor sleep and sleep-related disorders have had a significant economic impact, costing businesses and the healthcare system billions of dollars annually [7].

Alongside the lack of sleep, Americans have struggled to engage in the recommended amount of daily exercise. The 2015 National Health Interview Survey found that, from 1997 to 2015, over one-half of adults failed to meet the federal Physical Activity Guidelines for aerobic physical activity and only one-fifth satisfied the federal guidelines for both aerobic and muscle-strengthening activity [8]. One explanation may stem from the increasing proportion of Americans who do not prioritize leisure-time physical activity. From 1988 to 2010, one study reported that the number of women who do not exercise recreationally jumped from 19.1% to 51.7% while the fraction of their male counterparts rose from 11.4% to 43.5% [9]. Considering that Booth et al. describe exercise as the “primary prevention against 35 chronic health conditions,” especially cardiovascular disease and related disorders, this drastic reduction in leisure-time physical activity may contribute to the substantial prevalence of lifestyle diseases throughout American society [10, 11].

It should be of little surprise then that Americans who both lack proper sleep and fail to engage in regular exercise vastly increase their risk for chronic illness. Is it possible there is a link between these two behaviors? Since 2011, a significant amount of research has been aimed at understanding the physiology of sleep and the interrelationship between sleep and exercise. In addition, numerous studies have examined whether interventions involving increased physical activity impact sleep. The purpose of this report is to summarize the most recent literature exploring (i) how different modalities of exercise influence the subjective and objective qualities of sleep and (ii) the impact sleep quality and duration have on exercise performance. Additionally, this review will examine the physiological factors by which sleep is mediated and discuss why these factors may be so important in understanding sleep as a biological process.

## 2. Materials and Methods

### 2.1. Protocol and Registration

This review was conducted following the PRISMA statement for reporting systematic reviews and meta-analyses; it was not registered a priori.

### 2.2. Eligibility Criteria

The participants, interventions, comparisons, outcomes, and study design (PICOS) framework was utilized in the identification of concepts pertinent to the research question and was critical to the facilitation of the search process.

### 2.3. Population

This review analyzed several health populations and clinical populations. Healthy populations included adolescents, young adults, adults, and older adults without sleep disorders. Clinical populations included those with high blood pressure, obesity, diabetes, rheumatoid arthritis, insomnia, sleep apnea, and women in the postpartum period.

### 2.4. Intervention

Studies were included if they possessed either an objective (polysomnography, actigraphy, and accelerometry) or subjective (Pittsburg Sleep Quality Index, another self-report, and proxy-report) measure of sleep and an exercise intervention that followed the guidelines recommended by the American College of Sports Medicine.

### 2.5. Comparison

Various exercise interventions were used. A control group was not required for inclusion.

### 2.6. Outcome

Several sleep quality indicators were used based on the literature, expert consensus, and the importance of understanding the multiple factors influencing the quality and duration of sleep.

### 2.7. Study Design

Randomized controlled trials and observational studies were primarily considered. Studies were subdivided by population for whom the research was intended. A minimum number of participants were not required for either type of study.

### 2.8. Information Sources and Search Strategy

The electronic search strategy was created and completed by a single researcher, and the results were reviewed by the other members of the research team. To fulfill the purpose of this systematic review, a computerized literature search was performed over the course of two weeks utilizing PubMed and Google Scholar for articles indexed between January 2013 and March 2017. This narrow scope was used to emphasize recent investigations published in the last few years. Keywords used in various combinations were “sleep,” “exercise,” “regulation,” “loss,” “aerobic,” “heart rate variability,” “resistance,” “deprivation,” and “performance”.

### 2.9. Study Selection

Titles and abstracts of potentially relevant articles were screened. Articles were included based on relevance to the research question. This was determined according to the abstract, reference list of included articles, and other relevant reviews. Published peer-reviewed original manuscripts were eligible for inclusion. Studies were included if they were published in English.

### 2.10. Data Collection Process

A data extraction form was created and piloted. Extraction was completed manually to spreadsheet software (Excel; Microsoft Corporation, Redmond, WA). The investigators were not blinded to the authors or journals when extracting and analyzing the data.

### 2.11. Data Items

Important study features, such as publication year, study design, age and health profile of participants, measure of sleep and health outcomes, measure of fitness, results, and confounders, were extracted. The principle summary measures were difference in means and strength of correlation which were interpreted as improving sleep, worsening sleep, or having no significant impact.

## 3. Results

**Figure 1:**
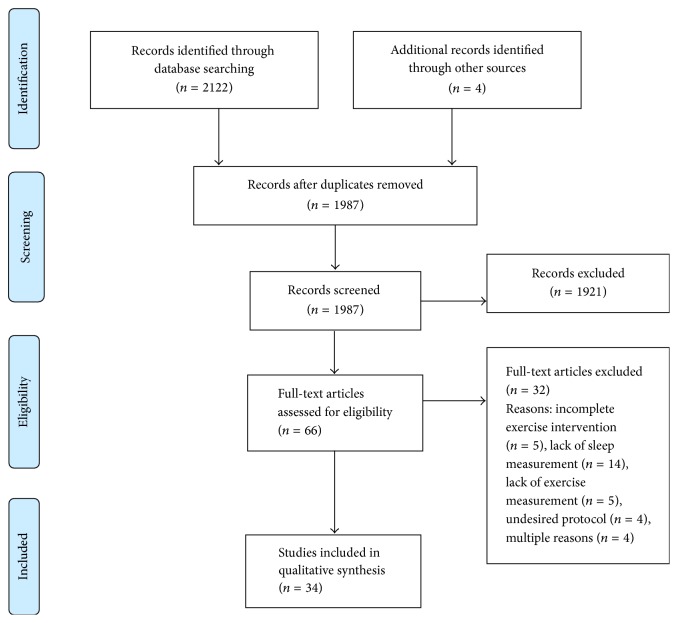
Preferred Reporting Items for Systematic Reviews and Meta-Analyses (PRISMA) flow diagram.

As shown in Figure 1, a total of 2122 records were identified from database searches. Four additional articles were identified from the reference list searches by the researcher. After removing duplicates, 1987 records remained. Once titles and abstracts were screened, 66 full-text articles were obtained for further review and 34 articles met the inclusion criteria. Characteristics of these studies are provided in Table 1. Reasons for excluding articles included incomplete exercise intervention (*n* = 5), lack of sleep duration or quality measurement (*n* = 14), improper measurement (*n* = 5), and undesired intervention protocol (*n* = 4). Some studies were excluded for multiple reasons (*n* = 4).

## 4. Discussion

### 4.1. The Physiology of Sleep

Although there is significant research surrounding sleep and exercise as they affect one another in multiple, diverse populations, the specific physiological factors by which the two interact are still undefined. Several hypotheses have been set forth and subsequent studies rely heavily upon them to base findings. Lack of sleep or experimental sleep restriction has been found to impair cognitive performance, mood, glucose metabolism, appetite regulation, and immune function [13]. Petit et al. suggest sleep is a glycogenetic process that replenishes glucose stores in neurons while the awake cycle is designed for repetitive glycogen breakdown [14]. This conclusion may indicate that the process of sleep affects the brain at an endocrine level independent of the hormonal regulation of metabolism and waste removal at the cellular level. Furthermore, this cascade of effects has been suggested to be the possible result of body temperature changes, cytokine concentration changes, increased energy consumption/metabolic rate, central nervous system fatigue, changes in mood and anxiety symptoms, changes in heart rate and heart rate variability, changes in growth hormone secretion, changes in brain-derived neurotrophic factor secretion, improved fitness level, and changes in body composition [15].

Abel et al. propose a different underlying function of sleep: to aid in the encoding and consolidation of memories [16]. The authors suggest that patterns of neural oscillations observed during sleep stimulate the neurobiological processes associated with synaptic plasticity and long-term potentiation. Physiologically, sleep is an internally and externally controlled process structured by an interaction of the circadian clock and homeostatic mechanisms [14]. Harp explains that photic and nonphotic entrainment of the central peripheral molecular machinery constitutes the external factors [17]. The internal factors include intrinsic molecular circadian clock mechanisms that moderate metabolic circadian rhythms. Specifically, these molecular mechanisms entail the positive and negative transcription feedback loops that produce proteins within a cell [14]. These regulating mechanisms function to classify sleep into two distinct states: rapid eye movement sleep (REM sleep) and non-REM sleep. These represent two of the three vigilance states, with wakefulness being the third. Non-REM sleep is subdivided into three stages, N1, N2, and N3. N1 is the stage that lies between wake and sleep. N2 represents a slightly deeper stage of sleep, with a higher auditory arousal threshold and changes in brain activity. N3 is also called deep sleep or slow-wave sleep due to the low-frequency, high-amplitude brain activity recorded by EEG during this stage [14]. REM sleep has important metabolic consequences due to the reported increase in metabolic rate and glucose utilization during REM sleep [18].

### 4.2. Sleep Recommendations throughout the Lifespan

To fully understand the issue at hand surrounding sleep and the lack thereof in the US population, it is important to recognize the current guidelines set forth by the National Sleep Foundation in Table 2 [12]. These guidelines are consistent with the American Academy of Sleep Medicine and Sleep Research Society's Joint Consensus Statement for healthy adults and with the Canadian 24-Hour Movement Guidelines for Children and Youth [1, 19]. It is important to note, however, that these recommendations can lack substantial empirical evidence and tend to change over time [20]. The basis for these guidelines stems from research suggesting that deviation from said recommendations may result in adverse health outcomes while adherence to them offers potential benefits that “far exceed the potential risks” [1, 19]. Some of the adverse health outcomes linked to sleeping less than the recommended amount include weight gain and obesity, diabetes, hypertension, heart disease and stroke, depression, and increased risk of death [21–25]. Additionally, chronic lack of sleep increase the potential for impaired immune function, increased pain, impaired performance, increased errors, and greater risk of accidents [1]. Recent evidence suggests a substantial portion of the population averages significantly less than the recommended duration of sleep per night indicating an obvious need to change the current sleeping habits of Americans [2]. Despite this negative trend, only recently has there been an exponential rise of research dedicated to investigating the causes behind lack of sleep and the interventions necessary to encourage greater duration and improve sleep quality.

### 4.3. Physical Activity and Exercise as It Pertains to Sleep throughout the Lifespan

In a variety of conditions such as cardiovascular disease, type 2 diabetes, depression, some cancers, and arthritis, physical activity and exercise are advocated as effective interventions for the treatment of disordered sleeping [26–28]. How exercise should be administered and when it should be practiced is still under investigation, but there are several findings of significance that support the use of exercise as a means to improve sleep quantity and quality throughout the lifespan.

One study demonstrated that twelve weeks of exercise training increased sleep duration and variables of sleep quality in adolescents [18]. These investigators found exercise training to decrease NREM stage N1 (very light sleep) while increasing REM sleep, sleep continuity, and sleep efficiency when using polysomnography. Conversely, Williams et al. found that as children's intensity of daily physical activity increased from light to moderate to vigorous, sleep duration decreased [29]. One possible explanation for this negative correlation involves the time utilized for exercise interfering with time available to sleep [29]. Another reason for these results could be tied to the methodology of the study, where it was suggested that physical activity and sleep duration should have been recorded over the course of several days and then averaged as opposed to measuring sleep following a single day of physical activity. Interestingly, the researchers stated that physical activity promotes improved sleep quality and efficiency rather than prolonged sleep duration [29]. In a different investigation of children's physical activity level and sleep, no significant associations were observed between the amount of light or moderate-vigorous exercise and total sleep time or sleep efficiency [30].

Several studies of young adults, primarily from college campuses, also reveal mixed effects of exercise on sleep. Variations in the methods of these investigations make it difficult to compare the findings across studies; however, it is worth discussing the differences as they may allude to further understanding of the sleep-exercise relationship. A review by Lang et al. explored these various differences in methodology and found that participants who engaged in high levels of physical activity were more likely to experience better sleep quality [31]. Yamanaka et al. evaluated the acute effects of daily aerobic exercise in young adult males over the course of six nights in a living facility using plasma melatonin, rectal temperature, polysomnography, and heart rate variability as various physiological measures [32]. These investigators reported that daily exercise of moderate intensity had differential effects on circadian melatonin rhythm, rectal temperature during nocturnal sleep, sleep stages, and heart rate variability depending on the time of day the exercise is performed. The interpretation of these results suggests that the timing of exercise is important for sleep quality. The authors concluded that exercise earlier in the day may improve the quality of nocturnal sleep owing to the fact that exercise stimulates the sympathetic nervous system. To improve sleep quality, Yamanaka et al. suggest enhancing parasympathetic activity by allowing time for the stimulation of the sympathetic nervous system to diminish.

Contrary to these findings, Alley et al. found that the timing of resistance exercise did not significantly affect total or REM sleep the following evening [33]. However, the investigators did conclude that, regardless of the time of day, engaging in resistance exercise did improve sleep quality. Specifically, they reported that variations in the timing of resistance exercise instead affected aspects of sleep such as sleep onset latency (SOL) or wake time after sleep onset (WASO). For instance, morning exercise was found to significantly improve the time required to fall asleep, and evening exercise was found to significantly reduce WASO. In a related study with adults, Fairbrother et al. compared SOL, WASO, and the number of times participants woke up during sleep after morning, afternoon, and evening exercise [34]. The investigators discovered that these sleep parameters were at their lowest after bouts of morning physical activity.

However, Brand et al. echoed the results of Alley et al.: they found exercise an hour and a half before bedtime to be associated with increased deep sleep and decreased REM sleep [35]. More specifically, individuals with greater self-perceived exertion during exercise had decreased light sleep and increased deep sleep compared with those who reported less self-perceived exertion [35]. The same investigators summarize their findings by stating that self-perceived exercise exertion and objectively assessed sleep are positively associated, meaning that the encouragement of exercise as part of daily physical activity likely benefits the objective aspects of sleep.

While the aforementioned studies primarily observe the acute effects of exercise on the following night of sleep, Harp evaluated the chronic effects of exercise on sleep in young adults [17]. Subjects in this investigation engaged in a 15-week aerobic exercise intervention and completed sleep quality questionnaires, such as the Pittsburg Sleep Quality Index (PSQI), at the start and end of the study [36]. This methodology allows for the analysis of sleep as a long-term beneficiary of regular exercise. Harp found that age, gender, and body composition are significantly related to sleep quality [17]. Importantly, participants classified as overweight or obese were found to experience poorer sleep quality than those of leaner body composition. However, the author did not find 15 weeks of exercise to change sleeping patterns for the majority of the participants. Instead those who were considered overweight/obese before the study showed improved sleep quality, likely the result of a decrease in body fat percentage from participating in regular exercise.

In adults, the effects of chronic and acute exercise on sleep become more resolute. In a meta-analysis by Kredlow et al., both acute and regular exercise were found to have a positive effect overall; however, this effect was small and often varied in strength depending on the component of sleep analyzed [15]. Acute exercise was reported to have a small effect on factors such as total sleep time, slow-wave sleep, SOL, and decreased REM sleep. Regular exercise was found to have moderate and strong positive effects on overall sleep quality while exhibiting moderate-to-largely strong positive effects on all subscales of the PSQI. Furthermore, chronic exercise can increase total sleep time and sleep efficiency to some degree and have a small-to-moderate effect on SOL [15].

One of the few experimental studies involving adults tested the chronic effects of exercise by combining a twice-a-week, six-week aerobic training program with daily physical activity [37]. This study also employed the commonly used PSQI to assess sleep quality and found a positive linear relationship between the global score and daily physical activity measured by step count. An increase in physical activity duration also produced better sleep quality scores [37]. The results of this study support the use of long-term exercise programming as an intervention for poor sleep quality in adults. It also lends support to the hypothesis that overall fitness is an indicator of sleep quality. Several of the following studies evaluate this hypothesis.

In a large-scale observational study, Wennman et al. examined the relationship between sleep and different motivations for exercise such as for leisure, occupational purposes, and transportation [38]. They discovered that individuals who slept best tended to engage in higher amounts of leisure physical activity while those who performed higher levels of occupational physical activity or no exercise at all tended to sleep worse. Dishman et al. analyzed the relationship between sleep quality and measured cardiorespiratory fitness [39]. Finding that odds of sleep complaints increased by 1.7% in men and 1.3% in women for each minute of decline in treadmill performance time using the Balke protocol, the authors suggest that enhancing cardiorespiratory fitness can be a useful intervention for improving sleep. A similar study by Strand et al. echoes these conclusions: the investigators found an inverse relationship between cardiorespiratory fitness, also measured by maximum oxygen uptake (VO_2max_) and symptoms associated with insomnia in a sample of over 3,000 Norwegian participants [40]. These results were further supported by two separate analyses of exercise levels and the incidence of insomnia symptoms in cohorts of over 12,000 [41] and 450,000 Chinese citizens [42]. Both studies found that decreased physical activity led to an increased risk for insomnia.

Other studies that indicate the need for bettering fitness to improve sleep quality include those of Farnsworth et al. and McClain et al. [43, 44]. The former team of investigators found that individuals with low levels of sedentary behavior, obesity, and physical activity were not significantly associated with developing a sleep disorder. Meanwhile those with low levels of sedentary behavior and physical activity but high levels of obesity had an elevated risk of a sleep disorder [43]. The significance of this finding is that key measures of fitness and body composition may be associated with risk of sleep disorders and therefore poor sleep quality. By improving body composition, Farnsworth et al. found that sleep quality improved as well. Interestingly, McClain et al. concluded that sedentary behavior and physical activity were not necessarily associated with sleep quality for all populations [44]. The investigators indicated that the relationship between physical activity and self-reported sleep duration is age- and sex-dependent. This explains why young or older adults may see benefits to sleep quality while middle-aged adults may not.

A final article analyzing the effects of exercise upon sleep in adults provides the most relevant information to healthy individuals who participate in a variety of daily activities throughout the day. Wennman et al. collected data regarding employment, sleeping patterns, napping patterns, and leisure-time physical activity from a national health survey seeking to study cardiovascular disease risk factors [38]. The survey answers allowed the investigators to statistically evaluate the interrelationship between physical activity and sleep. Upon evaluation, the results suggest that higher leisure-time physical activity was correlated with better sleep. This is interesting because it suggests time be set aside in every adult's day to engage in leisurely physical activity as opposed to what may be occupational physical activity. This emphasizes a need for exercise in an adult population to produce what Wennman et al. observed to be “longer, sufficient sleep”—sleep of higher quality and duration.

Again, as aging continues, research strongly points to the validation of exercise and daily physical activity to improve sleep. In older adults, the chronic effects of exercise encourage better sleep quality as do the acute effects of a single, moderate-to-vigorous intensity exercise session [45–49]. Only one investigation did not support the interrelationship between acute exercises and sleep quality; however, the participants were diagnosed with insomnia prior to testing [50]. The authors of this study noted in the study that the presence of an insomnia diagnosis should be considered when observing the effects of exercise on sleep.

Another common trend in research involving older adults is the modality and intensity of the exercise performed. Both Melancon et al. and Wang and Youngstedt used moderate-intensity aerobic exercise as an intervention, while Gambassi et al. employed a comprehensive resistance training program [51]. Siddarth et al. tested using aerobic exercise in one group and mind-body exercise, that is, yoga and Tai Chi, in the other group [48]. Du et al. used Tai Chi exclusively as an intervention [45]. All of these forms of exercise produced better sleep quality as measured by PSQI global scores or wrist actigraphy but Siddarth et al. found that mind-body exercise showed significantly better mood, mental health, and sleep compared to individuals participating in aerobic exercise [48]. This finding elicits a need to further understand the physiological mechanisms by which sleep interacts with exercise. Dzierzewski et al. suggest that mood regulation and anxiety and arousal are proponents that may impact sleep heavily in this population [46]. This may explain results of Siddarth et al. given the nature of mind-body exercise which focuses on being mindful of one's emotions and energy while simultaneously emphasizing relaxation and the release of tension through incorporating a blend of aerobic and resistance movements at a moderate intensity. It appears then that mind-body exercise may assist in alleviating symptoms of anxiety, arousal, or poor mood [45].

### 4.4. Exercise and Sleep in Special Populations

The previously mentioned studies concern exercise as it affects sleep in healthy populations throughout the lifespan. We also reviewed studies investigating this relationship in special populations. The first of these is athletes. Although this population is often seen as the epitome of health, athletes are less prone to see the beneficial effects of exercise on sleep if poor sleep quality exists. Mounting evidence shows that, due to a variety of reasons, such as a demanding training schedule, precompetition anxiety, and extensive traveling, athletes often experience sleep deprivation that in turn hinders their performance [52–54]. Several studies reported that one night of sleep deprivation can result in metabolic irregularities, such as decreased plasma lactate concentration as well as increased creatine phosphokinase and myoglobin levels, after a bout of exercise the following morning [55, 56]. To mitigate these consequences, Nédélec et al. suggest that athletes consistently employ sleep hygiene strategies—limiting exposure to electronic devices prior to bed, going to sleep in darkness, and waking up in natural light—to improve sleep quality the night before and after a competition [57].

As previously discussed, a significant reason why exercise might be so beneficial in young and middle-aged adults is due to the potential relationship between fitness and sleep. Athletes, if training and eating properly, should have already achieved a high level of fitness therefore exposing a difference in the interrelationship. Suppiah et al. examined the effects of different intensities of exercise in adolescent athletes [58]. The authors found that a group of athletes who engaged in a low-intensity sport, bowling, exhibited significant differences in EEG sleep patterns following days of training compared to a different group of athletes who practiced a high-intensity sport, badminton. Specifically, Suppiah et al. concluded that the athletes in the high-intensity group achieved a greater amount of deep sleep and decreased WASO than the low-intensity group [58]. However, in a related study by the same investigators comparing high- and low-intensity athletes, contrasting results were obtained. Adolescent sprinters did not experience significant differences in the amount of deep sleep or WASO compared to their counterparts who practiced shooting [59]. Similar conclusions were echoed by Harris et al. who compared sleep outcomes between elite adolescent athletes and age-matched controls [60]. No significant differences in sleep quality or WASO were observed, but athletes did report increased total sleep time on weekdays as well as increased sleep efficiency and decreased SOL on weekends. The findings from all three investigations support the idea that exercise intensity and sleep may influence each other differently within athletes compared to the general population. One challenge for elite athletes is that they cannot be subjected to much higher-intensity exercise every day to improve sleep quality as their training intensity is typically near their maximum capacity. Killer et al. subjected cyclists to nine consecutive days of intensified training to test the effectiveness of altering periodization and instead found that sleep quality decreased significantly and progressively throughout the period of training [61]. Further increasing the intensity of training in athletes would only subject them to higher risk of fatigue and overuse injury. Therefore, other methods are necessary. Halson suggests a series of nutritional recommendations for athletes trying to maximize sleep quality and quantity [13]. She concluded that (i) high-protein diets can improve sleep quality, (ii) high-fat diets can negatively affect total sleep time, (iii) sleep quantity may be disrupted if total caloric intake is decreased, and (iv) tryptophan, which may be consumed from turkey or pumpkin seeds, can improve SOL and sleep quality. These findings suggest that nutrition may be a more effective and necessary adaptation to an athlete's lifestyle to improve sleep quality and quantity. Marshall and Turner recommend sleep hygiene strategies as a means to improve these measures in athletes [62]. These strategies, which are simple and easy to maintain, yet rooted in the physiological mechanisms surrounding sleep, can be utilized by both athletes and nonathletes to improve sleep quality and quantity.

The second special population entails those with a diagnosed health disorder or health condition that has been observed to negatively influence sleep quantity or quality. These conditions include systemic hypertension, obesity, diabetes, rheumatoid arthritis, insomnia, sleep apnea, and women in the postpartum period. Again there is a growing consensus that exercise will benefit sleep for those experiencing one of these conditions. The following studies are a small representation of the health conditions associated with poor sleep and exercise intervention.

We have already discussed that one of the potential benefits of exercise on sleep is the improvement of body composition. Increased sleep disturbance is often seen with a diagnosis of obesity or type 2 diabetes mellitus [63]. Nam et al. analyzed the effects of weight loss brought about by a nutritional and exercise program versus a nutritional program alone to identify what factors might be associated with sleep disturbances [64]. Each group was subjected to a six-month program designed to result in the same amount of caloric expenditure, allowing for the observation of chronic effects of exercise and diet. Following the six-month program, the investigators observed similar changes to body weight, total abdominal fat, aerobic capacity (VO_2max_), and sleep disturbances in the two groups. They concluded that abdominal fat reduction and improvement of depressive symptoms would be most effective for reducing sleep disturbance [64]. Interestingly, these results differ from those of Iftikhar et al. who performed a meta-analysis of the effects of exercise training on sleep apnea [65]. The latter investigators found that the severity of obstructive sleep apnea, a common sleep disturbance, was not reduced by a decrease in body weight but was improved by exercise training. This study suggests that exercise may directly be responsible for sleep quality improvement instead of a change in body composition that impacts sleep apnea, a conclusion also supported by a different meta-analysis by Aiello et al. [66]. Since sleep apnea can be caused by anatomical features of the upper airway that are not necessarily related to body weight or body composition, it is possible that many patients continue to experience airway obstruction during sleep even when body weight is reduced or body composition is improved. Kline found exercise training to reduce sleep-disordered breathing severity in another meta-analysis [67]. The discrepancy in these findings is likely related to the fact that multiple factors contribute to airway obstruction during sleep.

Strand et al. found a moderate inverse and graded association between insomnia symptoms and cardiorespiratory fitness [40]. This means that, to some degree, sleep disorders may be related to lower fitness levels. Another study examined biomarkers in a cohort of sleep-disordered individuals diagnosed with major depressive disorder [68]. After a 12-week aerobic exercise intervention, participants exhibited reductions in brain-derived neurotrophic factor, interleukin-1*β*, and hypersomnia. This information elucidates a plausible mechanism for how exercise may indirectly influence sleep and suggests that those with sleep disorders may benefit from engaging in increased physical activity. Both Strand et al. and Kline observe that the relationship between a sleep disorder and physical activity is bidirectional [40, 67]. What may start as a sleep disorder results in greater fatigue throughout the day and thus lowers the likelihood of exercising [40]. Engaging in physical activity would increase cardiorespiratory fitness and thus improve sleep disorders including sleep apnea and insomnia. The reduction of these symptoms with exercise could then cause a responsive improvement to physiological and metabolic changes such as body temperature, heart rate, metabolic rate, activity of the hypothalamic-pituitary-adrenal axis, hypertension, and diabetes [40].

In patients with rheumatoid arthritis, sleep disturbance is more common than in the general population [26]. This observation may be related to pain, depression, lack of exercise, restless legs, and corticosteroid use. Durcan et al. recruited 78 patients diagnosed with rheumatoid arthritis and assigned 40 of those individuals to a 12-week home exercise program incorporating stretching, strength, and walking exercise [26]. They found that patients in the exercise program experienced significant reductions in pain, stiffness, and functional disability. Compared to patients in the control group, patients who received the exercise intervention showed a significant change in fatigue levels and sleep quality. In their investigation, Løppenthin et al. suggest that exercise may decrease symptoms of poor sleep by decreasing symptoms of depression and by interacting with the two-process model of circadian and homeostatic regulation [27]. Because of the multifactorial nature of rheumatoid arthritis, that is, how it negatively affects a patient both physically and psychosocially, engaging in exercise may not only improve sleep quality but also mitigate some of the symptoms [26, 27].

A final example of the influence of sleep on exercise is found in postpartum women. Ashrafinia et al. found that eight weeks of Pilates exercise 72 hours to one week after delivery significantly improved subjective sleep quality, SOL, daytime dysfunction, and global PSQI scores compared to the control group [69]. Such findings suggest that exercise, in this case in the form of Pilates, could be beneficial to the improvement of sleep quality in postpartum women. Ashrafinia et al. also suggest the physical and mental factors associated with Pilates may be beneficial to sleeping as they evoke a series of beneficial effects including increasing the body's core strength, circulation, and relaxation while decreasing musculoskeletal pains [69].

### 4.5. Limitations

The objective of this article was to review existing literature and to explore the relationship between sleep and exercise as exercise may contribute to greater duration and better quality of sleep. The identified research indicates exercise is beneficial for sleep duration and quality but there are differences across age groups in terms of the magnitude of this benefit. It is clear that additional research is necessary particularly because of the variance of these findings between various age groups, study populations, type of exercise intervention, and physiological reasons for why the effects of exercise upon sleep might occur.

## 5. Conclusion

Presently in American society, an inability to sleep and sleep well is a pervasive health concern. Despite the magnitude of this problem, the physiological function of sleep in regulating normal hormonal and metabolic processes is not fully recognized. However, that does not dismiss the mounting evidence that physical exercise is an effective intervention for those who do not experience adequate sleep quantity or quality. This review summarizes some of the findings as they apply to Americans throughout the lifespan and some of the special conditions in which the sleep-exercise interaction is critical. Further research needs to explore the biological mechanisms that modulate the dynamic interplay between these two aspects of human lifestyle.

## Figures and Tables

**(a):** 

Reference	Study design	*N*	Study conditions	Age group (range or mean ± SD)^*∗*^	Health condition	EX intervention
Aiello et al. (2016)	Meta-analysis	180	EX versus control	Adults (32–54)	OSA	AE
						RT

Alley et al. (2015)	Single group	24	EX versus control	YA (18–25)	Healthy	RT

Ashrafinia et al. (2014)	Open trial	80	EX versus control	YA, adults (18–35)	Postpartum	Pilates

Baron et al. (2013)	Single group	11	EX versus control	OA (≥55)	Insomnia	AE

Brand et al. (2014)	Observational	52	Moderate versus vigorous EX	YA (19.70 ± 0.30)	Athletes	Various sports

Chen et al. (2017)	Observational	12728	Different levels of physical activity and smoking	YA, adults, OA (18–≥65)	Healthy, smokers	Various

Dishman et al. (2015)	Observational	8523	Different levels of cardiorespiratory fitness	YA, adults, OA (20–85)	Healthy	AE (VO_2peak_)

Du et al. (2015)	Meta-analysis	460	EX versus control	OA (65–75)	Healthy	Tai chi
					SC	

Durcan et al. (2014)	RCT	78	EX versus control	Adults, OA (59 ± 12)	RA	AE
						RT

Dzierzewski et al. (2014)	Single group	79	EX versus control	Adults, OA (63.58 ± 8.66)	Healthy	—

Erlacher et al. (2015)	Open trial	98	EX versus control	YA, adults, OA (22–77)	SC	AE

Fairbrother et al. (2014)	Single group	20	EX during different times of day	Adults (30–60)	Prehypertension	AE

Gambassi et al. (2015)	Open trial	16	EX versus control	OA (65 ± 3)	Healthy	RT

Harp (2015)	Single group	2027	Pre- versus post-EX	YA (21.8 ± 5.0)	Healthy	AE

Harris et al. (2017)	Observational	74	Athletes versus controls	Adolescents (16.7 ± 0.72)	Healthy, athletes	Various

Iftikhar et al. (2014)	Meta-analysis	129	EX versus control	Adults (49.1 ± 8.5)	OSA	AE, RT

Killer et al. (2015)	Single group	13	Pre- and post-EX	YA (25 ± 6)	Athletes	—

Kredlow et al. (2015)	Meta-analysis	2863	Acute EX versus control	YA, adults, OA (18.3–88.5)	Healthy, athletes, SC	AE, RT
			Chronic EX versus control	YA, adults, OA (18.3–88.5)	Healthy, SC	AE, Tai chi, Yoga

Lang et al. (2016)	Meta-analysis	16549	High versus low EX versus control	Adolescents, YA (14–24)	Healthy, athletes	—

Løppenthin et al. (2014)	RCT	44	EX versus control	YA, adults, OA (18–70)	RA	AE

Melancon et al. (2015)	Single group	13	EX versus control	Adults, OA (57–70)	Healthy	AE

Mendelson et al. (2016)	Open trial	40	EX versus control	Adolescents (14.5 ± 1.5)	Obese, healthy	AE, RT

Nam et al. (2016)	Open trial	77	Diet + EX versus diet alone	Adults, OA (35–65)	Type 2 diabetes	—

Rethorst et al. (2015)	Open trial	126	High versus low EX	YA, adults, OA (18–70)	Major depression	AE

Siddarth et al. (2014)	Observational	42	Mind-body EX versus AE	Adults, OA (64.6 ± 13.6)	Healthy	Yoga, Tai chi, AE

Strand et al. (2013)	Observational	3489	Different levels of cardiorespiratory fitness	Adults, OA (51.9 ± 12.4)	Healthy	AE (VO_2peak_)

Suppiah et al. (2015)	Observational	11	Low- versus high-intensity EX versus control	Adolescents (14.8 ± 0.9)	Athletes	Bowling (low intensity), badminton (high intensity)

Suppiah et al. (2016)	Observational	29	Low- versus high-intensity EX versus control	Adolescents (14.7 ± 1.3)	Athletes	Shooting (low intensity), sprinters (high intensity)

Vincent et al. (2017)	Single group	65	Different levels of physical activity	Children (8–11)	Healthy	Various

Wang and Youngstedt (2014)	Single group	15	Light versus moderate EX versus control	Adults, OA (60–74)	Healthy	AE

Wennman et al. (2014)	Observational	6414	Leisure versus commuting versus occupational EX versus control	YA, adults, OA (25–74)	Healthy	Various

Williams et al. (2014)	Observational	234	Light versus moderate-vigorous EX versus control	Children (3–7)	Healthy	Various

Yamanaka et al. (2015)	Single group	22	Morning versus evening EX versus control	YA (22 ± 1.8)	Healthy	AE

Zheng et al. (2017)	Observational	452024	Different levels of physical activity	Adults, OA (30–79)	Healthy	Various

**(b):** 

Reference	Volume/frequency	Duration	Sleep characteristics assessed	Outcome measure	Result
Aiello et al. (2016)	30–150 min/day, 2–7 days/week	2–6 months	DS	ESS	Decrease
	—	—	Nocturnal hypopnea	AHI	Decrease

Alley et al. (2015)	30 min/day	3 days	TW	sEEG	Decrease

Ashrafinia et al. (2014)	30 min/day, 5 days/week	8 weeks	SQ	PSQI	Increase
			SOL	PSQI	Decrease

Baron et al. (2013)	30 min/day, 3 days/week	16 weeks	SQ, SOL, WASO	PSQI, WA	No change
			TST, SE	WA	Increase

Brand et al. (2014)	≥70 min/day, 2-3 days/week	—	Amount of deep sleep	sEEG	Increase
			SOL, TW, WASO	sEEG	Decrease

Chen et al. (2017)	Self-reported	2 weeks	Insomnia	ICD-9-CM codes	More active → decreased risk

Dishman et al. (2015)	Tested once/2-3 years, 4 times	8–12 years	Sleep disturbances	Medical consultation	More fit → decrease

Du et al. (2015)	20–60 min/day, 2–5 days/week	8–26 weeks	SQ, TST	PSQI	Increase
			SOL, DS	ESS, PSQI	Decrease

Durcan et al. (2014)	30–60 min/day, 5 days/week	12 weeks	SQ	PSQI	Increase
	2-3 days/week		Fatigue	FSS	Decrease

Dzierzewski et al. (2014)	20 min/day	18 weeks	SQ	Sleep diary	Increase
			SOL	Sleep diary	No change
			WASO	Sleep diary	Decrease

Erlacher et al. (2015)	60 min/day, 3 days/week	6 weeks	TW, WASO	PSQI	Decrease
			SOL, TST	PSQI	No change

Fairbrother et al. (2014)	30 min/day	4 days	TST	sEEG	No change
			TW, SOL, WASO	sEEG	Lowest during AM EX

Gambassi et al. (2015)	2 days/week	12 weeks	SQ, SE	PSQI	Increase
			SOL, TST, DS	PSQI	No change

Harp (2015)	30 min/day, 3 days/week	15 weeks	SQ, TST	PSQI	No change

Harris et al. (2017)	Self-reported	1 week	SQ, WASO	Sleep diary	No difference
			SOL		Lower for athletes on weekends only
			SE		Higher for athletes on weekends only
			TST		Higher for athletes on weekends only

Iftikhar et al. (2014)	15–90 min/day, 3–5 days/week	12–24 weeks	DS	ESS	Decrease
			Nocturnal hypopnea	AHI	Decrease
			SE	ESS	Increase

Killer et al. (2015)	≥2 hours/day, ≥3 days/week	18 days	TW	WA	Increase
			SQ, SE	WA	Decrease
			SOL	WA	No change

Kredlow et al. (2015)	1 session/day	1 day	TST, SE	PSQI, sEEG, PSG	Increase
			TW	PSQI, sEEG, PSG	No change
			SOL, WASO	PSQI, sEEG, PSG	Decrease
	—	2–52 weeks	SQ, TST, SE	PSQI, sEEG, PSG, WA	Increase
			SOL	PSQI	Decrease

Lang et al. (2016)	—	1–105 days	SQ, SE	PSQI, sEEG, Sleep logs	Increase
			Insomnia	ISI	Decrease

Løppenthin et al. (2014)	20–30 min/day, 2-3 days/week	8 weeks	SQ, TST, SOL	PSG, PSQI	In progress
			DS	ESS	In progress

Melancon et al. (2015)	1 hour/day, 3 days/week	16 weeks	WASO	PSG, PSQI	Decrease
			SQ, SOL, TST, SE	PSG, PSQI	No change

Mendelson et al. (2016)	180 minutes/week	12 weeks	SQ, TST, SE	PSG	Increase
			SOL	PSG	No change

Nam et al. (2016)	3 days/week	6 months	Sleep disturbances	JHSS	Decrease in both groups

Rethorst et al. (2015)	4 KKW	12 weeks	SQ	IDS-C	Increase
	16 KKW				

Siddarth et al. (2014)	1 hour/day, 1-2 days/week	—	Sleep disturbances	PROMIS-SDSF	Fewer in mind-body group

Strand et al. (2013)	Tested once	—	Insomnia	HUNT-3 questionnaire	More fit → decrease

Suppiah et al. (2015)	16 hours/week	1 week	DS	KSS, PDSS	No difference
			TST	WA	Greater during control
			WASO	sEEG	Lower in high group
			TST	sEEG	No difference

Suppiah et al. (2016)	16 hours/week	1 week	TST, WASO	sEEG	No difference
			TW, TST, WASO	WA	Greater during control
			SE	WA	No change

Vincent et al. (2017)	Various	8 days	TST, SE	TA	No change

Wang and Youngstedt (2014)	~1 hour/week	2 weeks	TW, WASO	WA	Lower in moderate group
			TST	WA	No change

Wennman et al. (2014)	Various	—	SQ, TST	Questionnaires	Greatest in those with high leisure EX

Williams et al. (2014)	Data collected for ≥5 days every 6 months	4 years	TST	Sleep log	More active → decrease

Yamanaka et al. (2015)	2 hours/day, 4 days/week	1 week	SOL, WASO, TST, SE	PSG	No change

Zheng et al. (2017)	Self-reported	1 year	Insomnia	Questionnaires	More active → decreased risk

*N* = number of participants; RCT = randomized controlled trial; EX = exercise; YA = young adults; OA = older adults; OSA = obstructive sleep apnea; SC = participants with sleep complaints; RA = rheumatoid arthritis; AE = aerobic exercise; RT = resistance training; VO_2peak_ = peak oxygen uptake; KKW = kilocalories per kilogram of body weight per week; DS = daytime sleepiness; TW = times woken from sleep; SQ = sleep quality; SOL = sleep-onset latency; TST = total sleep time; WASO = wake time after sleep onset; SE = sleep efficiency; ESS = Epworth Sleepiness Scale; AHI = Apnea-Hypopnea Index; PSQI = Pittsburgh Sleep Quality Index; WA = wrist actigraphy; sEEG = sleep electroencephalography; FSS = Fatigue Severity Scale; PSG = polysomnography; ISI = Insomnia Severity Index; JHSS = Johns Hopkins Sleep Survey; AM = ante meridiem (morning); IDS-C = clinician-rated Inventory of Depressive Symptomatology; PROMIS-SDSF = Patient Reported Outcomes Measurement Information System: Sleep Disturbance Short Form; HUNT-3 = Helseundersøkelsen i Nord-Trøndelag; KSS = Karolinska Sleepiness Scale; PDSS = Pediatric Daytime Sleepiness Scale; TA = triaxial accelerometer; ICD-9-CM = International Classification of Diseases, Ninth Revision, Clinical Modification

^*∗*^Age groups were based on the demarcations outlined in Table 2  [12].

**Table 2:** The National Sleep Foundation's recommended amount of sleep per age group [12].

Newborns	0–3 months	14–17 hours
Infants	4–11 months	12–15 hours
Toddlers	1-2 years	11–14 hours
Preschoolers	3–5 years	10–13 hours
School-age Children	6–13 years	9–11 hours
Teenagers	14–17 years	8–10 hours
Younger adults	18–25 years	7–9 hours
Adults	26–64 years	7–9 hours
Older adults	65+ years	7-8 hours
