# Corrigendum to “Interrelationship between Sleep and Exercise: A Systematic Review”

**DOI:** 10.1155/2017/5979510

**Published:** 2017-10-16

**Authors:** Brett A. Dolezal, Eric V. Neufeld, David M. Boland, Jennifer L. Martin, Christopher B. Cooper

**Affiliations:** ^1^Exercise Physiology Research Laboratory, Departments of Medicine and Physiology, David Geffen School of Medicine at UCLA, Los Angeles, CA, USA; ^2^VA Greater Los Angeles Healthcare System, Geriatric Research, Education and Clinical Center, North Hills, CA, USA; ^3^Department of Medicine, David Geffen School of Medicine at UCLA, Los Angeles, CA, USA

In the article titled “Interrelationship between Sleep and Exercise: A Systematic Review” [1], there was an error in Section  4.4 (Exercise and Sleep in Special Populations). The text reading “Several studies reported that one night of sleep deprivation can result in metabolic irregularities, such as decreased plasma lactate concentration as well as increased creatine phosphokinase and myoglobin levels, after a bout of exercise the following morning [55,56]” should be corrected to “Several studies reported that one night of sleep deprivation can result in metabolic irregularities, such as decreased plasma lactate concentration as well as increased creatine phosphokinase and myoglobin levels, after a bout of exercise the following evening [55,56].”
